# Accuracy of reirradiation dose constraints for the mandible and carotids

**DOI:** 10.1016/j.phro.2025.100897

**Published:** 2025-12-21

**Authors:** Sara Bornedal, Jeehong Lee, Tim Melhus, Anna Embring, Eva Onjukka

**Affiliations:** a Department of Nuclear Medicine and Medical Physics, Karolinska University Hospital, Stockholm, Sweden; b Department of Radiotherapy, Karolinska University Hospital, Stockholm, Sweden; c Department of Oncology Pathology, Karolinska Institutet, Stockholm, Sweden

**Keywords:** Reirradiation, Head and neck cancer, Dose constraints, Deformable image registration, Accumulated dose

## Abstract

When reirradiation dose constraints are derived using accumulated dose, the underlying image registrations contribute to the uncertainty. We performed a structure-based evaluation of deformable image registrations, to estimate the uncertainty in previously published dose constraints related to carotid blowout and osteoradionecrosis after head and neck reirradiation. With the workflow of the current analysis, the uncertainty was small in the majority of the cases (<4 Gy in accumulated equivalent dose in 2-Gy fractions), but with substantial outliers resulting from anatomical alterations. Our previously suggested dose constraints appear to be reliable with regard to the underlying image registrations.

## Highlights

• Evaluation of intensity-based deformable image registrations for reirradiation.
• Generally subvoxel geometrical uncertainty for organs at risk in the head and neck.
• Outliers among carotids due to anatomical changes and poor image quality.
• Provides confidence in reirradiation dose constraints for head and neck cancer.

## Graphical abstract

**Figure:**
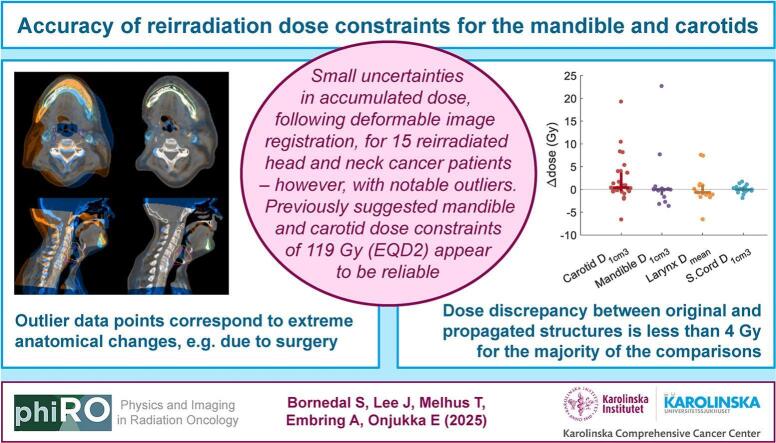

## 1 Introduction

The challenges in planning safe and effective reirradiation for recurrent- or second primary cancer have received increasing attention [1], [2], [3]. The uncertainties involved propagate to the interpretation of outcome data for organ-at-risk (OAR) dose response [4], resulting in scarce and uncertain dose constraints for the reirradiation setting [5]. According to a patterns-of-care survey, the pelvis and the head and neck are the most common sites of reirradiation, after the brain [5]. In previous work, we studied the accumulated dose to OAR, in relation to severe late side effects, for reirradiated head and neck cancer (HNC) patients receiving at least 100 Gy accumulated dose, in equivalent dose in 2-Gy fractions (EQD2) [6]. The results provided much needed support for previously proposed dose constraints regarding carotid blowout [7] and osteoradionecrosis (ORN) [8], which were based on very limited evidence. The dose summation was performed using dose distributions in EQD2 after deformable image registration (DIR) [9], according to consensus recommendations [10]. Even so, geometrical uncertainties are unavoidable in such a cohort, considering the anatomical changes associated with the temporal separation between the treatments (up to nearly 15 years), differences in treatment setup and the mix of external-beam radiotherapy and brachytherapy.

DIR is an effective means of mapping dose in the context of dose accumulation, and it seems to obtain a higher degree of consistency compared to alternative methods [4]. It has been implemented in clinical practice by some centres, but the majority still rely on rigid registration for dose mapping [5]. Commonly, concerns about the uncertainties of DIR prevent its adoption [11]. The accuracy of a DIR algorithm can only be assessed under controlled conditions, e.g. using physical or digital phantoms [12]. There is no standard procedure for estimating the uncertainty in real patient cases [13], but an indication is often sought through studying the consistency [4], [12], [14], e.g. between centres or between algorithms.

In the current analysis, we applied a structure-based evaluation of the DIR uncertainty in a cohort of reirradiated HNC cases, as an indication of the accuracy in this anatomical region at reirradiation, for one commercially available DIR algorithm. The specific aim was to estimate the uncertainty in accumulated dose metrics used to derive previously published dose–response relationships.

## 2 Material and methods

### 2.1 Dataset and dose accumulation

A sample of fifteen out of 54 cases from the study on side effects after HNC reirradiation [6], [9] were included in this analysis. These included the eleven most recent cases (one of which had ORN), and four additional cases with ORN and/or carotid blowout. The study was approved by the Swedish Ethical Review Authority.

The dose accumulation for the reirradiation study, and the corresponding image registrations, were performed in a research version (2023BR) of RayStation (Raysearch Laboratories, Stockholm, Sweden), after importing the treated plans from the original treatment-planning system. There were two computed tomography (CT) image sets per patient (primary treatment and reirradiation). For each patient, the most recent image set (used for treatment planning at reirradiation) was appointed reference. For all the sets of treatment plans, an initial rigid registration was performed, followed by an intensity-based DIR. The rigid registration prioritised agreement in the dose overlap region, with manual adjustments after the initial automatic registration. The DIR algorithm used was ANACONDA [15], which is proprietary to RaySearch. Further details about the performed image registrations are given in the supplementary materials.

Each dose distribution was converted to EQD2 in RayStation, using *α/β* = 3 Gy. The EQD2 distribution corresponding to the primary treatment was propagated to the reference image set, followed by an accumulation of the primary- and the reirradiation EQD2 distributions in the reference (reirradiation) image set.

### 2.2 Estimation of image registration uncertainty

Structure-based evaluation of the image registrations was performed. The supplementary materials include details on the OAR delineations. As carotid blowout and ORN were among the severe side effects in our cohort, and dose constraints were suggested in relation to these endpoints, the carotids and mandible were of particular interest (although not all cases of ORN developed in the mandible). The spinal cord and the larynx were also included, as relevant structures for dysphagia and myelopathy, respectively. Some cases of severe dysphagia were observed in the original cohort, but no myelopathy.

A quantitative evaluation of the geometrical uncertainty was performed by comparing the structures propagated from the primary treatment to the reference treatment-planning CT scan and the corresponding structures delineated directly in the reference image. A set of commonly used structure-based metrics with complementing strengths were calculated (e.g. [13]): the mean- and maximum distance to agreement, respectively (*DTA_mean_* and *DTA_max_*), and the Dice similarity coefficient (DSC) [16]. These chosen metrics and their application are discussed in the supplementary materials.

Finally, the uncertainty in accumulated dose was estimated by evaluating the consistency between a dose metric of interest for the propagated structures, and for the structures delineated directly in the reference image set, respectively. For the carotids, the near maximum dose (D_1cm3_, i.e. the minimum dose to the hottest 1 cm^3^ of the volume) was considered, for the left and right carotid independently. This metric was used also for the mandible and the spinal cord, while for the larynx, the mean dose (D_mean_) was considered more relevant. Notably, this uncertainty estimation did not evaluate the actual dose mapping error, i.e. the variation in accumulated dose per voxel resulting from different image registrations.

## 3 Results

The visual evaluation of the registrations, using blending of the fused CT images and structure comparison, showed only small deviations (example in Fig. S1 in the supplementary materials), despite relatively large anatomical changes from the primary treatment to the reirradiation, in some cases.

With regard to the geometrical uncertainty, the results for the mandible and the spinal cord were excellent, with a median *DTA_mean_* of 0.6 mm (inter-quartile range (IQR) = 0.5–1.2 mm) and 1.0 mm (IQR = 0.8–1.2 mm), respectively (Table 1, Table S1 and Figs. S2–S3). The carotids and the larynx showed a slightly higher median *DTA_mean_* of 1.6 mm (IQR = 1.4–2.1 mm) and 1.8 mm (IQR = 1.7–2.2 mm), respectively. In addition to the overall discrepancies, larger local discrepancies were common, as quantified by *DTA_max_*. The median *DTA_max_* ranged from 6.8-8.0 mm for all OAR except the spinal cord which had a value of 4.2 mm (IQR = 3.0–5.1 mm). There were 7 cases of a structure exceeding 15 mm in *DTA_max_*.

**Table 1:** Mean- and maximum distance to agreement in millimeter.

	Carotid_L		Carotid_R		Mandible		Larynx		Spinal cord	
Case	*DTA_mean_*	*DTA_max_*	*DTA_mean_*	*DTA_max_*	*DTA_mean_*	*DTA_max_*	*DTA_mean_*	*DTA_max_*	*DTA_mean_*	*DTA_max_*
1	3.1	12.4	3.1	12.8	3.1	51.7	2.3	13.8	1.2	5.1
2	1.7	6.2	2.3	7.5	0.3	5.2	1.9	6.4	0.9	4.7
3	1.8	9.1	1.4	6.1	0.6	6.8	1.6	7.8	0.8	2.9
4	1.5	7.6	1.0	4.5	0.3	4.3	1.8	9.4	1.0	4.4
5	1.4	9.4	1.6	7.6	0.3	4.3	1.5	7.1	0.8	2.9
6	1.2	5.7	0.9	3.4	1.3	11.9	1.8	7.7	1.3	3.1
7	3.9	17.3	5.2	16.2	0.5	8.6	5.7	19.4	1.2	5.3
8	1.3	7.8	1.4	7.3	0.4	3.0	0.9	4.0	1.0	7.0
9	1.9	7.6	0.9	4.7	1.6	12.6	1.8	7.5	0.7	2.4
10	1.5	7.2	2.7	10.7	0.5	6.8	2.2	9.5	0.8	3.5
11	1.6	9.9	2.1	12.3	0.6	22.2	2.7	9.4	1.2	5.1
12	1.6	5.7	1.6	5.9	1.0	3.2	−*	−*	0.8	3.1
13	2.1	10.2	2.1	15.3	2.9	58.2	1.8	8.4	1.4	4.2
14	3.1	9.3	2.1	11.8	0.9	8.7	2.1	8.1	2.1	18.7
15	1.3	7.8	1.2	4.7	0.5	6.0	1.4	5.9	0.0	0.3

Median	1.6	7.8	1.6	7.5	0.6	6.8	1.8	8.0	1.0	4.2
IQR	1.5–2.0	7.4–9.7	1.3–2.2	5.3–12.0	0.5–1.2	4.8–12.3	1.7–2.2	7.2–9.4	0.8–1.2	3.0–5.1
* Missing value due to laryngectomy										
IQR = inter-quartile range										

For the carotids, all except 6 (out of 30) propagated structures showed a discrepancy of 4 Gy (in EQD2) or less from the reference (delineated directly in the treatment-planning CT); this number was 2/15 for the mandible, 3/14 for the larynx (one patient had had a laryngectomy) and 0/15 for the spinal cord (Table 2 and Fig. S4). Since most of the outliers accumulated in a few patients (see supplementary materials), the discrepancy in the dose metric was 4 Gy or less (in EQD2) for 8/15 patients, and despite these outliers, the median value of the absolute difference in the full cohort was: 1.0 Gy (IQR = 0.4–3.9 Gy) in carotid D_1cm3_, 0.2 Gy (IQR = 0.1–3.0 Gy) in mandible D_1cm3_, 1.1 Gy (IQR = 0.8–1.7 Gy) in larynx D_mean_, and 0.5 Gy (IQR = 0.3–1.1 Gy) in spinal cord D_1cm3_. Please refer to the supplementary materials for details about outliers, a correlation analysis between geometric and dosimetric uncertainty metrics and the commissioning of the DIR tool.

**Table 2:** Accumulated dose (EQD2, Gy) to OAR computed in the reference structure (Ref.) and the propagated structure (Prop.), respectively.

	Carotid_L D_1cm3_		Carotid_R D_1cm3_		Mandible D_1cm3_		Larynx D_mean_		Spinal cord D_1cm3_	
Case	Ref.	Prop.	Ref.	Prop.	Ref.	Prop.	Ref.	Prop.	Ref.	Prop.
1	97.7	97.3	8.2	9.2	133.2	133.3	12.5	10.8	17.5	18.8
2	52.3	52.4	97.0	105.4	71.7	71.7	39.5	38.7	34.4	35.5
3	93.0	92.5	64.6	68.2	121.0	121.2	48.2	47.6	36.6	36.5
4	47.2	47.5	12.8	12.8	110.7	107.9	1.2	2.1	21.5	21.3
5	94.5	94.7	13.4	13.3	131.0	131.0	25.0	23.8	35.9	34.8
6	80.8	81.7	66.0	66.4	113.7	113.6	43.6	37.1	31.6	31.1
7	87.3	95.5	98.3	108.7	90.3	90.9	73.9	81.3	49.2	48.6
8	40.8	42.5	119.5	119.0	112.8	111.2	24.1	25.0	44.2	44.7
9	113.8	117.5	74.5	72.8	115.9	123.6	43.9	42.3	35.4	35.7
10	51.5	52.9	66.4	70.4	62.9	62.9	44.9	43.8	19.9	19.6
11	74.0	73.6	49.4	49.7	103.5	99.9	61.1	68.7	45.9	45.9
12	126.0	125.1	76.3	76.2	112.8	109.6	−*	−*	38.9	39.0
13	95.4	114.6	81.9	75.4	99.7	122.4	46.2	47.4	35.7	33.8
14	117.7	115.7	65.0	69.1	121.1	120.8	51.5	50.6	32.6	34.3
15	67.8	73.2	17.4	18.4	72.2	72.2	1.1	1.3	23.2	23.7
* Missing value due to laryngectomy										

## 4 Discussion

With the commercial solution for DIR used in the current analysis, we found the geometrical uncertainties in DIR to be generally small, in the setting of HNC reirradiation. The uncertainty in accumulated OAR dose was around 2–4 Gy (EQD2), with a median below 1 Gy (EQD2). This corresponds to 2–3 % of the mandible- and carotid dose-constraint of 119 Gy (EQD2). However, outliers can be expected for OAR like the carotids (low CT contrast, and a branching structure which can adapt its shape to the surrounding anatomy), and in cases of steep dose gradients or extreme anatomical changes from the primary setting to the reirradiation setting. The carotids appear vulnerable to such factors (see discussion about outliers in supplementary materials) and need to be evaluated carefully in the fused images before relying on DIR-based dose accumulation.

Quantitatively, the *DTA_mean_* was typically around 2 mm or less, which indicates good conformity, as this is less than the voxel size and acceptable by previously suggested standards [19]. The median discrepancy in the dose metric was only up to 1.1 Gy, for all OAR analysed. Still, for the carotids, D_1cm3_ deviated more substantially in a few cases. When retrospectively reviewing the two cases of carotid blowout reported in our previous work [6], the carotids were confirmed to have been integrated in a relatively uniform dose overlap region, and they were clearly visible without interference from dental artefacts. Thus, the suggested dose constraint for carotid blowout is unlikely to suffer from major image-registration related uncertainty. The relevance of the geometric uncertainty metrics is discussed in the supplementary materials.

For the mandible, the deviation in D_1cm3_ was less than 4 Gy for all cases except two, indicating a low uncertainty except in cases of surgical resection. One of the patients with ORN in our previous work was such a case, but the ORN was located in the maxilla (with bone D_1cm3_ exceeding mandible D_1cm3_, and the hotspot located in the maxilla). Therefore, the uncertainty in the previously suggested dose constraint for ORN is expected to be reliable from a geometrical consideration. Even though ORN developed in the mandible in only one of the cases from that study, and the dose was thus evaluated in the general bone structure, the uncertainty can be expected to be similar or lower for bone compared to the mandible specifically. Similarly, the uncertainty in the accumulated dose to the spinal cord was very low, likely aided by the rigid anatomy and the clear image contrast for the vertebrae. For a discussion of the reliability and generalisability of the dosimetric uncertainty metrics, please see the supplementary materials.

In the setting of reirradiation, the uncertainty in the accumulated dose to OAR needs to be considered when applying a dose constraint [4]. In regions of poor agreement between fused images, it can be unsafe to consider DIR-based accumulated dose. Here, the best approach may be to consider a worst-case scenario, based on e.g. the maximum dose in EQD2 for the respective treatments, either globally or regionally. Where DIR-based dose accumulation is performed, planning risk volumes should be used to avoid steep dose gradients adjacent to OAR sensitive to hot spots, similarly to the primary treatment setting. The size of the margin should then take the uncertainty in the DIR into account.

The methods used to manage the image-registration related uncertainty in the current analysis follow current recommendations [13], [17], [18], including commissioning of the DIR tools, and evaluation of both the geometrical and the dosimetric uncertainty. The analysis considers a relatively simplistic estimate of the dosimetric uncertainty, with a focus on the consistency of dose metrics of interest, rather than on the actual dose mapping error. This consistency was evaluated from a single accumulated dose distribution, comparing different versions of the OARs. The selected approach followed from the aim to verify previously published dose constraints. For extended discussions regarding strengths, weaknesses and interpretations of the results, please refer to the supplementary materials.

In conclusion, the geometric uncertainty of the current DIR methodology was in the order of the voxel size for most of the cases, and OAR. However, local uncertainties of 1–2 cm were not uncommon. Specifically, regarding our previously suggested dose constraints for carotid blowout and ORN, these appear to be reliable with regard to the underlying DIR, based on the magnitude of the uncertainty demonstrated in the current analysis, and specific evaluation of the reported cases of severe side effects.

## CRediT authorship contribution statement

**Sara Bornedal:** Conceptualization, Data curation, Formal analysis, Methodology, Supervision, Validation, Writing – review & editing. **Jeehong Lee:** Data curation, Investigation, Methodology, Writing – review & editing. **Tim Melhus:** Investigation, Methodology, Supervision, Writing – review & editing. **Anna Embring:** Conceptualization, Data curation, Investigation, Methodology, Project administration, Writing – review & editing. **Eva Onjukka:** Conceptualization, Data curation, Formal analysis, Funding acquisition, Investigation, Methodology, Project administration, Resources, Software, Supervision, Visualization, Writing – original draft, Writing – review & editing.

## Declaration of competing interest

The authors declare that they have no known competing financial interests or personal relationships that could have appeared to influence the work reported in this paper.
