# Supplementary material for: Accuracy of reirradiation dose constraints for the mandible and carotids
Source: Phys Imaging Radiat Oncol. 2025 Dec 21;37:100897. doi: 10.1016/j.phro.2025.100897 (PMC12799775; doi:10.1016/j.phro.2025.100897)
Supplement: Supplementary Data 1 [file mmc1.pdf]

Supplementary Materials

Supplementary figures and data tables

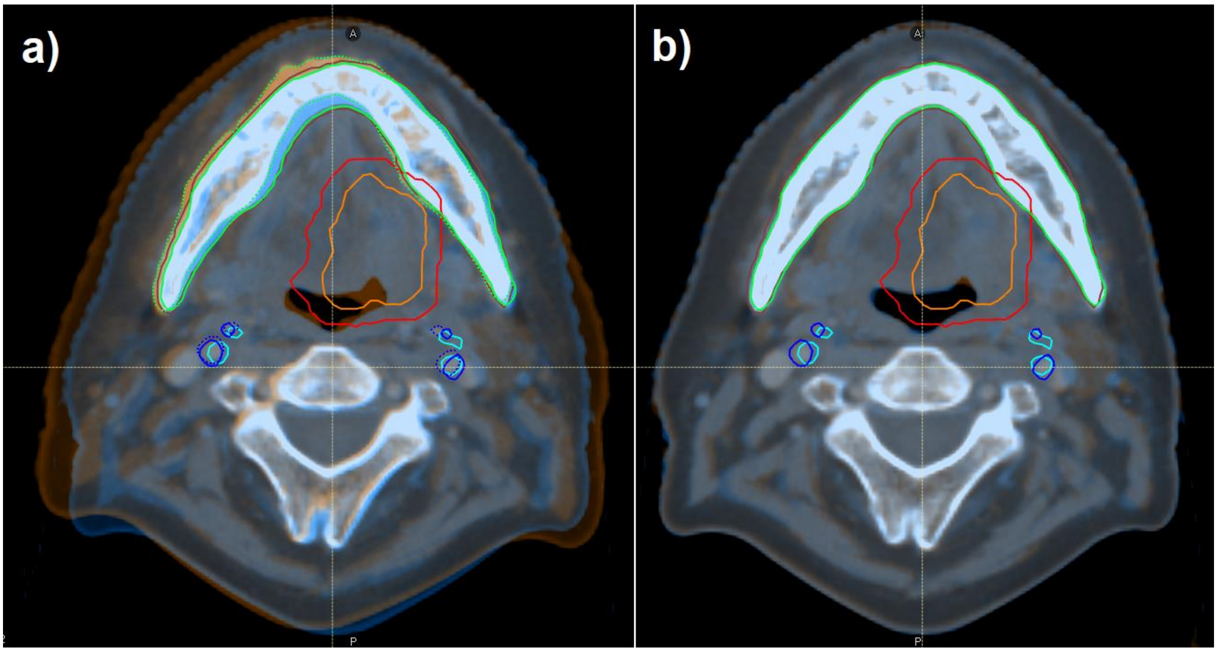

Figure S1. a) The rigid registration and b) the subsequent deformable registration, between the primary treatment-planning CT and the reirradiation treatment-planning CT, for one of the HNC cases of the study. The reirradiation CT (reference) is displayed in standard greyscale values, while the primary CT is tinted golden. The full lines represent structures delineated directly in, or propagated to, the reirradiation CT, while the propagated versions of the OAR in the primary treatment CT are dashed. Brown/green = mandible, cyan/dark blue = carotids, red = reirradiation PTV, orange = reirradiation CTV.

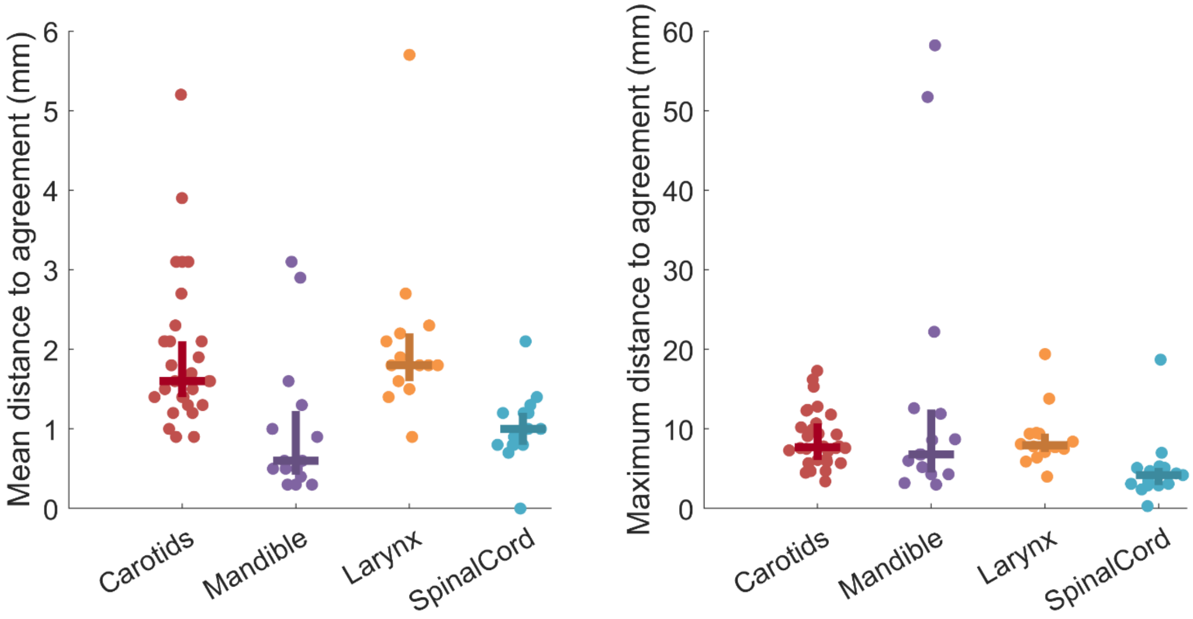

Figure S2. Mean- (left) and maximum (right) distance to agreement, for the evaluated OAR. The right and left carotid structures were considered as individual OAR. The lines indicate the median and IQR.

Table S1. Dice similarity coefficient.

Case	Carotid_L	Carotid_R	Mandible	Larynx	Spinal cord
1	0.25	0.33	0.84	0.81	0.79
2	0.55	0.45	0.96	0.85	0.87
3	0.35	0.54	0.92	0.85	0.85
4	0.61	0.68	0.95	0.84	0.83
5	0.65	0.58	0.96	0.87	0.87
6	0.56	0.70	0.84	0.82	0.79
7	0.32	0.08	0.93	0.59	0.80
8	0.62	0.57	0.94	0.92	0.84
9	0.38	0.67	0.82	0.85	0.88
10	0.46	0.27	0.94	0.81	0.84
11	0.54	0.51	0.92	0.75	0.79
12	0.43	0.43	0.88	-*	0.84
13	0.45	0.55	0.87	0.86	0.78
14	0.18	0.48	0.88	0.81	0.74
15	0.60	0.66	0.93	0.85	0.99
Median	0.46	0.54	0.92	0.84	0.84
IQR	0.37-0.58	0.44-0.62	0.87-0.94	0.81-0.85	0.79-0.86

*Missing value due to laryngectomy.

IQR = inter-quartile range

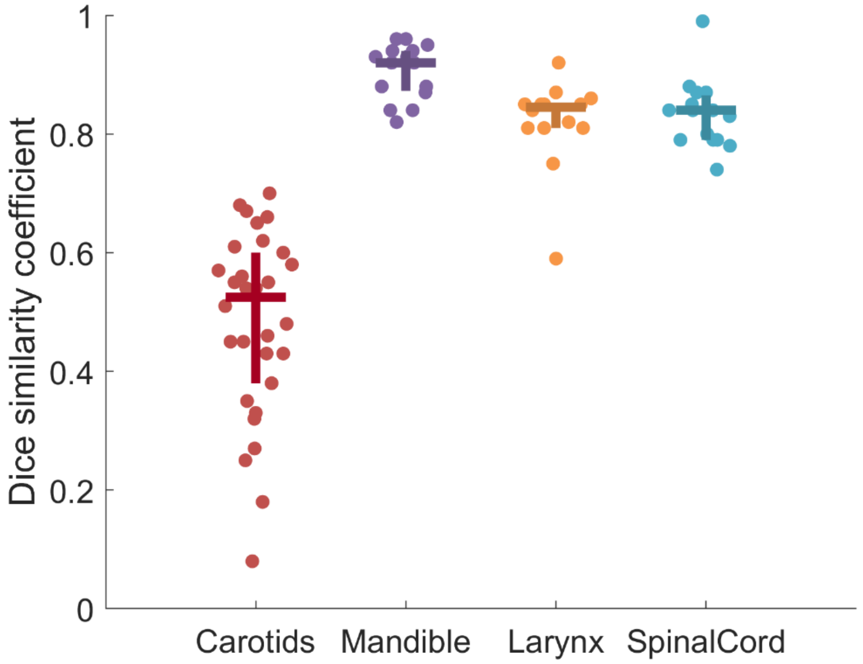

Figure S3. Dice similarity coefficient (DSC) for the evaluated OAR. The right and left carotid structures were considered as individual OAR. The lines indicate the median and IQR.

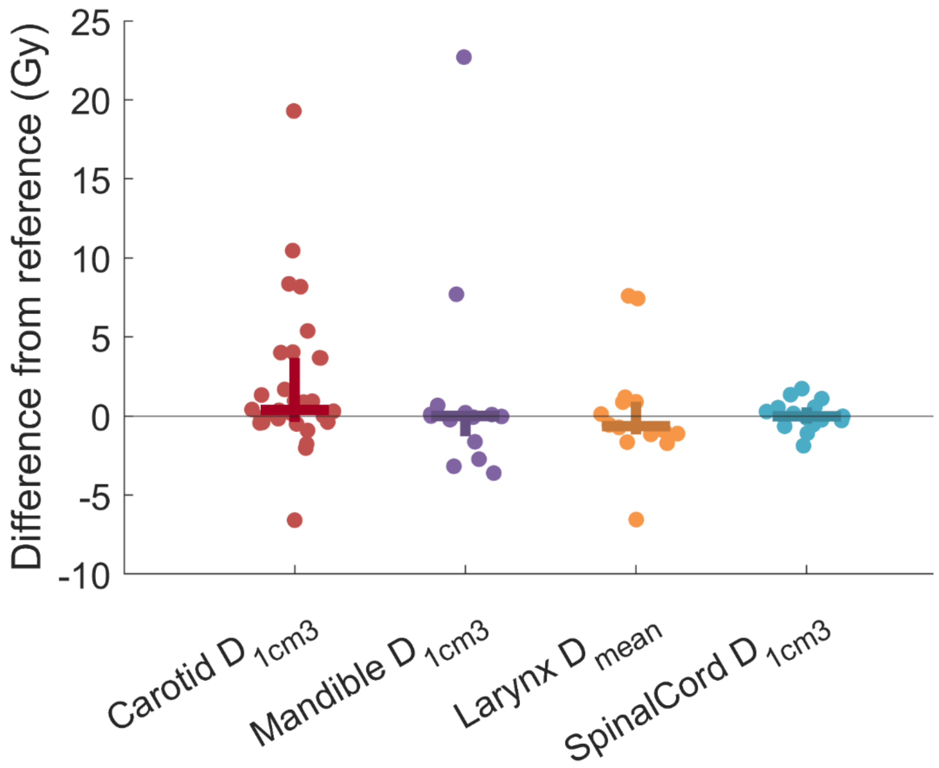

Figure S4. Difference in accumulated dose (in EQD2) for the structure propagated from the primary treatment-planning CT and the structure delineated directly in the reirradiation treatment-planning CT, respectively. The value of the metric for the structure delineated in the reference CT (i.e. the reirradiation CT) was subtracted from the value for the propagated structure. The near-maximum dose (D_{1cm3}) was considered for the carotids and the mandible, and D_{mean} for the larynx. The right and left carotid structures were considered as individual OAR. The lines indicate the median and IQR.

Extended discussion

Organ-at-risk delineation

The four organs-at-risk (OAR) were delineated for the purpose of this study, by one and the same experienced head and neck cancer (HNC) oncologist, in all image sets separately. In the case of the carotids and the spinal cord, the cranio-caudal extent of the delineation was matched in each image pair, by trimming the longer one while reviewing the structure in the fused images. Similarly, the cranial extent of the mandible was made pair-wise consistent. The common-, internal- and external carotid arteries were included.

Image registrations

In RayStation, the deformable image registration (DIR) algorithm, ANACONDA, uses a non-linear cost function and a normalised image intensity cross-correlation coefficient as similarity metric, combined with a grid regularisation term. By default, the registration is applied to the intersection of the external body structure from the two image sets. This region of interest was also applied in the current study. In contrast, previous investigators have raised the importance of applying a region of interest in the high-dose region to focus the DIR [1, 2]. However, visual inspection of the deformations confirmed smooth variations, promoted by the regularisation term in the ANACONDA optimiser [3]. We observed one case of peripheral distortion of the deformed image, affecting DTA_{max} for the spinal cord, but this was not close to the high-dose region. In addition, the initial rigid registration was

performed carefully for every case in the current study, before applying DIR, specifically focusing on the overlap region.

The reference image sets had a resolution of 1 mm x 1 mm in the transversal plane and a 2 mm slice thickness. The resolution of the dose grid was 2.5 mm in all dimensions; this resolution was also selected for the image registrations (the resolution of the deformation map can be modified by the user).

The performance of the image registration tools in RayStation was verified using the digital-phantom based commissioning procedure recommended by AAPM TG132 [4], including both the geometric- and the anatomic phantoms. Each rigid registration and each DIR was evaluated visually, and the Jacobian determinant was reviewed to detect any non-physical results (local values ≤ 0). The commissioning was successful; the image registration tools passed all tests. Also, for the current dataset including fifteen DIRs of reirradiation for HNC, the Jacobian determinant showed only positive values throughout the body volume, with no indication of non-physical results.

Due to technical problems, the original image registrations from the study on side effects after HNC reirradiation [5] were unavailable at the time of data processing, and new registrations were performed, using the same procedure. Only the four non-consecutive cases (with carotid blowout/osteoradionecrosis) were included with their original registrations.

We applied intensity-based DIR, rather than using the paired delineations of each OAR to perform a hybrid registration, which has been shown to improve DIR in applications for HNC [6]. The intensity-based DIR was chosen since the purpose of the study was to estimate the uncertainty in accumulated dose metrics reported in previous reirradiation studies [5, 7], where the accumulated OAR dose was evaluated based on delineations in the reference CT series (i.e. at reirradiation), using intensity-based DIR. Hybrid DIR cannot be performed where suitable paired delineations are not available, e.g. due to limitations in image quality in the primary treatment CT. Also, the hybrid-type algorithm seems to result in greater improvements in the pelvis than in the head and neck, when compared to intensity-based algorithms [6]. In line with this, our visual evaluation of the registrations typically indicated an uncertainty of a similar magnitude to the uncertainty which can be expected in the OAR delineations themselves.

Geometric uncertainty metrics

A selection of common metrics was used to evaluate the geometric uncertainty of the DIRs. While the DICE similarity coefficient (DSC) is widely reported in the literature it can act as a comparison metric, but its sensitivity to deviations depends on the size of the structure. Therefore, the use of DSC as a metric for image registration evaluation has been criticized [8]. Distance metrics like DTA_{mean} and DTA_{max}, on the other hand, provide intuitive measures of the uncertainty. They quantify the mean- and maximum distance, respectively, from each point on one version of the structure to the closest point on the other version of the structure. Thus DTA_{max} (also referred to as the Hausdorff distance) can reveal potentially important local deviations not captured by DTA_{mean}, while the overall agreement is better represented by the latter.

However, DTA_{mean} and DTA_{max} operate on point pairs in two versions of each structure, and where the proximal side of the other version of the structure is closer to points which should be compared to the distal side, they underestimate the deviation. For branching structures like the carotids, instances of comparisons to the wrong arm will occur, further underestimating the deviation for this structure. Great difficulty was sometimes experienced in delineating the carotids, especially in the primary treatment CT images, due to poor contrast or dental artefacts, implying that the low DTA_{mean} values could underestimate the actual uncertainty. On the other hand, DTA_{max} can give a relevant indication of the distances between thin structures with no overlap, which was the case in some slices for the carotids. The low DSC values for the carotids reflect the poor overlap between the propagated

structure and the reference structure, but they were likely exacerbated by the small diameter of the structure, where a relatively small distance to agreement can lead to a great loss of intersection between the two versions of the structure.

Correlation between geometric and dosimetric metrics

The Spearman rank correlation was evaluated between the absolute value of the dose metrics and each geometric uncertainty metric, respectively. The evaluation was performed separately per OAR as well as jointly for all. A p value of less than 0.05 was considered significant.

The dosimetric uncertainty (absolute deviation in the dose metric) was found to not correlate with any of the geometric uncertainty metrics, neither for each OAR separately, nor when considering all OAR jointly. This suggests a sensitivity in the dosimetric metrics to dose gradients, as previously observed when evaluating the uncertainty in accumulated dose to the mandible [9].

Analysis of outliers

For the mandible, there were two outliers with large DTA_{mean} and DTA_{max}; these were both due to surgical removal of part of the mandible between the two treatment courses. One of these patients developed ORN, although it was located in the maxilla. Similarly to the mandible, the two outliers in carotid DTA_{mean} were for a patient whose anatomy was greatly changed from surgery between the two treatment courses (Figure S5). For this case, the reirradiation anatomy presented a noticeably smaller patient volume in the dose overlap region, forcing the relative position of the carotids to shift and the larynx to shrink. Consequently, the same patient also presented as an outlier in DTA_{mean} for the larynx.

Four of the largest outliers for the carotid dose discrepancy corresponded to 2 cases, one discussed above (Figure S5) and one mentioned as having had part of the mandible resected, but here the uncertainty was also influenced by a very steep dose gradient and poor image quality of the primary treatment CT. Consequently, this patient was also an outlier for the mandible dose discrepancy. The difficult case from Figure S5 was also an outlier for the larynx dose discrepancy, while for the other two larynx outliers, the discrepancy was due to differences in neck flexing in the respective patient set-ups, which could result in large rotations of the cranial surface of the propagated structure.

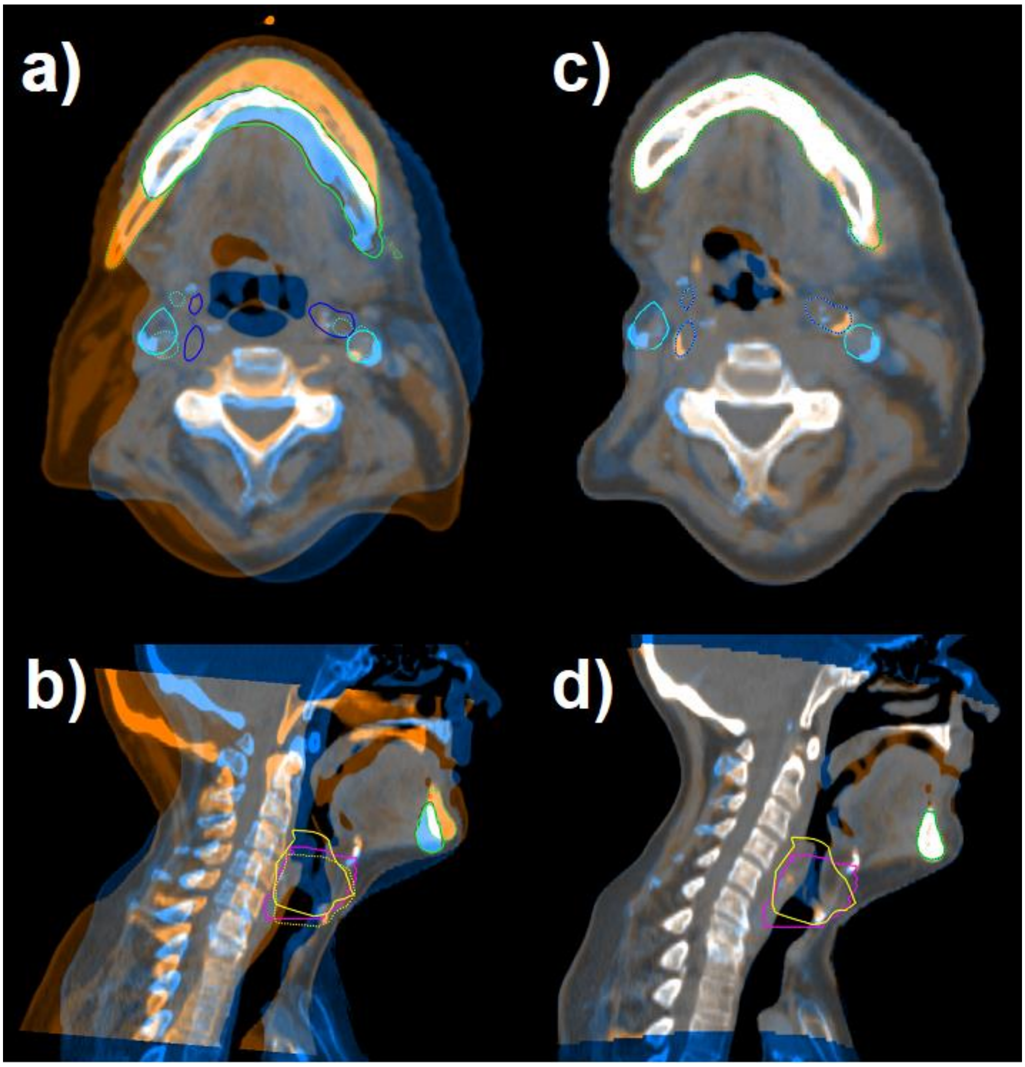

Figure S5. Challenging case of anatomical alteration due to surgery, resulting in a greater uncertainty in accumulated dose to the larynx and carotids. a-b) Initial rigid registration and c-d) DIR. The full lines represent structures delineated directly in, or propagated to, the reirradiation CT, while the propagated versions of the OAR in the primary treatment CT are dashed. Brown/green = mandible, cyan/dark blue = carotids, magenta/yellow = larynx.

Reliability and generalisability of the dosimetric uncertainty

We analysed the uncertainty in different dose metrics for different OAR (D_{1cm3} or D_{mean}). The type of dose metric considered may result in different uncertainties, with volumetric dose metrics appearing more robust [9]. There were cases where dental artefacts and poor tissue contrast challenged both the identification of the carotids, and the image registration in this area, leading to cases of large local geometric uncertainties. Since 1 cm^3 is a sizable proportion of the carotids, D_{1cm3} is somewhat insensitive to local deviations for this structure. These may be better captured by $D_{2\%}$ (the minimum dose to the hottest 2%), which in our cohort showed slightly greater deviations compared to D_{1cm3} (median = 2.0 Gy, IQR 1.0 - 4.3 Gy, data not shown).

The current analysis highlighted the impact of the geometric appearance of the structures on the dosimetric uncertainty. The small cross-section of the carotid and its adaptability to the surrounding anatomy seems to have limited the overlap between the two versions of the structure. Our results highlight the need for the carotids to be specifically evaluated with regard to dosimetric uncertainties on a case by case basis, e.g. as described by Nenoff et al. [2].

As a compact cylindrical structure, the definition of the larynx, on the other hand, depends strongly on the orientation of the CT slices in the anatomy, and differences in pitch can lead to notable differences at the orientation of the cranial/caudal planes. This can introduce partly artificial deviations between the structures. Frequently abutting the PTV, the steep dose gradient can make even the mean dose to the larynx uncertain.

In our cohort, more than four years had passed from primary radiotherapy to reirradiation in ten out of fifteen cases, the longest interval was nearly 15 years. At reirradiation, the anatomy had gone through surgery-, weight- and age-related alterations, and there was a lower image quality and image resolution for the very early treatments, providing a challenging task for the DIR algorithm. While reirradiation is becoming increasingly common, the patients in the original cohort were not treated according to a standardized protocol. Inherently, the disease, sensitivity of OAR and anatomical characteristics vary to a greater extent at reirradiation. Thus, the cohort of 15 cases (30 carotids, as a paired organ) in this analysis cannot capture all the variability which can be expected among patients who need reirradiation. On the other hand, the analysis reflects many of the challenges typically encountered.

References

[1] Murr M, Brock KK, Fusella M, Hardcastle N, Hussein M, Jameson MG, et al. Applicability and usage of dose mapping/accumulation in radiotherapy. Radiother Oncol 2023;182:109527. https://doi.org/10.1016/j.radonc.2023.109527.

[2] Nenoff L, Amstutz F, Murr M, Archibald-Heeren B, Fusella M, Hussein M, et al. Review and recommendations on deformable image registration uncertainties for radiotherapy applications. Phys Med Biol 2023;68:24TR01. https://doi.org/10.1088/1361-6560/ad0d8a.

[3] Weistrand O, Svensson S. The ANACONDA algorithm for deformable image registration in radiotherapy. Med Phys 2015;42:40–53. https://doi.org/10.1118/1.4894702.

[4] Brock KK, Mutic S, McNutt TR, Li H, Kessler ML. Use of image registration and fusion algorithms and techniques in radiotherapy: Report of the AAPM Radiation Therapy Committee Task. Med Phys 2017;44:E43–E76. https://doi.org/10.1002/mp.12256.

[5] Embring A, Onjukka E, Mercke C, Lax I, Berglund A, Bornedal S, et al. Re-Irradiation for Head and Neck Cancer: Cumulative Dose to Organs at Risk and Late Side Effects. Cancers 2021;13:3173. https://doi.org/10.3390/cancers13133173.

[6] Loi G, Fusella M, Lanzi E, Cagni E, Garibaldi C, Iacoviello G, et al. Performance of commercially available deformable image registration platforms for contour propagation using patient-based computational phantoms: A multi-institutional study. Med Phys 2018;45:748–57. https://doi.org/10.1002/mp.12737.

[7] Embring A, Onjukka E, Mercke C, Lax I, Berglund A, Bornedal S, et al. Overlapping volumes in re-irradiation for head and neck cancer - an important factor for patient selection. Radiat Oncol 2020;15:147. https://doi.org/10.1186/s13014-020-01587-3.

[8] Rohlfing T. Image Similarity and Tissue Overlaps as Surrogates for Image Registration Accuracy: Widely Used but Unreliable. IEEE Trans Med Imaging 2012;31:153–63. https://doi.org/10.1109/Tmi.2011.2163944.

[9] Hardcastle N, Vasquez Osorio E, Jackson A, Mayo C, Aarberg AE, Ayadi M, et al. Multi-centre evaluation of variation in cumulative dose assessment in reirradiation scenarios. Radiother Oncol 2024;194:110184. https://doi.org/10.1016/j.radonc.2024.110184.
